# A multidisciplinary self-management intervention among patients with multimorbidity and the impact of socioeconomic factors on results

**DOI:** 10.1186/s12875-019-0943-6

**Published:** 2019-04-22

**Authors:** Éric Contant, Christine Loignon, Tarek Bouhali, José Almirall, Martin Fortin

**Affiliations:** 1 Postgraduate student, Faculty of Medicine and Health Sciences, Université de Sherbrooke, Quebec, Canada; 2 Family Medicine Department, Université de Sherbrooke, Quebec, Canada; 3 Professor, Family Medicine Department, Université de Sherbrooke, Quebec, Canada; 4 Centre intégré universitaire de santé et de services sociaux du Saguenay–Lac-Saint-Jean, 305 St-Vallier, Chicoutimi (Québec), G7H 5H6 Canada

**Keywords:** Self-management, Multimorbidity, Primary care, Socioeconomic factors

## Abstract

**Background:**

Limited studies exist on successful interventions for patients with multimorbidity. Even more limited is the knowledge on how socioeconomic factors have an impact on these interventions. The objective of this study was to analyze the effect of a multidisciplinary self-management intervention among patients with multimorbidity and the impact of socioeconomic factors on the results.

**Methods:**

Secondary data analysis limited to multimorbid patients from of a pragmatic randomized trial evaluating an intervention that included patients (18 to 75 yrs.) from eight primary care practices in Quebec, Canada. The intervention included self-management support and patient-centred motivational approaches. Self-management was evaluated using the Health Education Impact Questionnaire (heiQ) which measures eight different domains. Changes in heiQ were analyzed following the three-month intervention with univariate and multivariate logistic regressions.

**Results:**

Participants with three or more chronic conditions (*n* = 281), randomized to intervention or control groups, were included in this analysis. The effect of the intervention on the likelihood of an improvement in self-management was significant in six heiQ domains in the univariate analysis (Odd ratio; 95% CI): *Health-directed behaviour* (2.03; 1.16–3.55), *Emotional well-being* (1.97; 1.05–3.68), *Self-monitoring and insight* (2.35; 1.02–5.40), *Constructive attitudes and approaches* (2.91; 1.45–5.84), *Skill and technique acquisition* (1.96; 1.13–3.39), and *Health services navigation* (2.52; 1.21–5.21). After controlling for age and gender the results remained essentially the same. After additional adjustments for family income, education and self-perceived financial status, the likelihood of an improvement was no longer significant in the domains *Emotional well-being* and *Self-monitoring and insight*.

**Conclusions:**

The intervention produced significant improvements in multimorbid patients for most domains of self-management. Socioeconomic factors had a minor impact on the results.

**Trial registration:**

ClinicalTrials.gov Identifier: NCT01319656

## Background

Multimorbidity, defined as the presence of multiple chronic conditions in an individual, [1, 2] is highly prevalent in primary care. [3, 4] However, despite its high prevalence and its important burden on patients’ health, there is limited evidence supporting care strategies for patients with multimorbidity. Clinical guidelines are mostly single disease oriented and they rarely address multimorbidity, leaving the clinician empty handed. [5, 6] A few guiding principles suggest getting a global understanding of the patient’s condition and social situation, exploring the patient preferences and priorities, and developing an individualized management plan [7–9].

One strategy that has been proposed for the management of chronic diseases is self-management support. That is, to encourage and support patients, to highlight their crucial role in maintaining health and function, as well as the importance of setting goals, establishing action plans, identifying barriers and solving problems to overcome them. [10] The positive effect of self-management support is known for single chronic diseases, [11] but little is known about the impact of such programs among patients with multimorbidity.

A systematic review of interventions in primary care and the community for the management of patients with multimorbidity suggested that interventions addressing risk factors or functional difficulties seemed more effective, but consideration of the impact of the socioeconomic factor was minimal [12]. It has been reported that multimorbidity occurs 10–15 years earlier in more socioeconomically deprived areas and is more prevalent in patients with lower socioeconomic status. [3, 13] Given the need to account for the heterogeneity of multimorbidity; the authors of the above-mentioned review suggested that interventions could have differential effects depending on the socioeconomic status of participants. [14] Recently, an intervention targeting multimorbid patients in high socioeconomic deprivation areas in Scotland used self-management to improve quality of life [15]. The results were positive and promising.

PR1MaC was a Canadian trial supporting self-management for patients with chronic diseases in primary care [16]. It was designed for patients with one or multiple chronic diseases and their risk factors. The intervention improved self-management in patients with chronic diseases. The purpose of the present study, nested in the PR1MaC trial, was to analyze the effect of a multidisciplinary self-management support intervention for patients with multimorbidity (3 or more chronic conditions), and to explore the effect of socioeconomic factors on the results of the intervention.

## Methods

### Settings and subjects

Our study analyzed secondary data from a pragmatic randomized controlled multidisciplinary intervention in patients with chronic diseases **(**PR1MaC Trial Registration: ClinicalTrials.gov Identifier: NCT01319656). [16, 17] The study took place in eight primary care practices in the Saguenay region of Quebec, Canada. Healthcare professionals recruited and trained by the research team, traveled from one site to the other to deliver the services in order to prevent differences in implementation between settings. Primary care providers from the eight practices referred patients 18 to 75 years to the research team to assess eligibility and obtain informed consent. Patients recruited had at least one of the following chronic conditions or risk factors: diabetes, cardiovascular disease, COPD, asthma, tobacco smoking, obesity, hyperlipidemia, prediabetes, sedentary lifestyles or any combination. Our secondary analysis only included patients with multimorbidity, defined as the presence of three or more of any chronic condition including at least one from this list.

Detailed information on the intervention and results are provided elsewhere. [16, 17] The intervention included self-management support with health education and a patient-centered motivational approach. After a preliminary clinical evaluation, an individualized intervention plan was designed by a trained nurse in collaboration with the patient. The plan could include encounters with one or more Chronic Disease Prevention and Management (CDPM) professionals in the disciplines of nursing, physical activity, nutrition, respiratory therapy, and smoking cessation therapy. The intervention plan could be further adapted by any participating professional in those disciplines. Regular contact with the CDPM professionals was maintained in order to ensure the reliability of the process. Each intervention was individualized, provided by the health professional at the clinic where the subject was recruited, and given over a three-month period at the most. The intervention consisted of at least three individual encounters.

### Randomized trial

Patients agreeing to participate completed an initial set of questionnaires at baseline (T1) including socio-demographic data. As participants were invited to receive the intervention in their usual clinic, blind randomization was not possible. Both patients and primary care providers knew who was involved in each group. Patients were randomized to receive the intervention immediately after baseline (group A: intervention group) or at the end of a three-month waiting period (group B: control group). The second questionnaire was completed after three months.

### Outcome

The impact of the educational activity was measured with the Health Education Impact Questionnaire (heiQ) [18, 19] that provides a broad profile of the potential impact of patient educational interventions. The heiQ evaluates eight different domains: 1) *Health directed behavior* (a change in lifestyle specifically related to healthy behaviors); 2) *Positive and active engagement in life* (embodies the notion of participants engaged in self-management/patient education programs or engaging in life-fulfilling activities); 3) *Emotional well-being* (a sense of individuals’ general emotional well-being and satisfaction with life); 4) *Self-monitoring and insight* (individuals’ ability to monitor their condition(s), and their physical and/or emotional responses that lead to insight and appropriate actions for self-management); 5) *Constructive attitudes and approaches* (how individuals view the impact of their condition(s) on their life); 6) *Skill and technique acquisition* (the knowledge-based skills and techniques that participants acquire or relearn to help them manage and cope with disease-related symptoms and health problems); 7) *Social integration and support* (the positive impact of social engagement and support that evolves through interaction with others and the impact that may arise from interaction with others sharing similar health-related life experiences); 8) *Health services navigation* (an individual’s understanding of and ability to confidently interact with a range of health organizations and health professionals). Each domain is standardized to range from 1 to 4. Baseline and follow-up data are compared to determine the achievement of meaningful changes in each domain (see ‘Data analysis’ for details). A validated French Canadian version of the questionnaire was used in the study. [20]

### Data analysis

We compared participant characteristics in the two groups using Student *t* test for continuous variables, and the chi-square test for categorical variables. The classical reliable change index, [21] in which each individual before-after difference in score is corrected by dividing the difference by the standard error of the difference, was used to evaluate meaningful individual changes in the heiQ domains. The primary endpoint of 3-month change in the heiQ domains was then called a ‘reliable improvement’ when the classical reliable change index was > 1.65 in a domain of the heiQ, as suggested by the developers of the heiQ.

There were only a few missing values in the three-month questionnaire (less than 5%). Therefore, we undertook a “complete case” analysis, which is almost equivalent to the intention-to-treat analysis without requiring strong imputation assumptions, which may be hard to justify. [22]

We developed univariate and multivariate logistic regression models to evaluate the association between the effect of the intervention and obtaining a reliable improvement in the heiQ domains. In a first model, we ran a univariate logistic regression with the presence or absence of a reliable improvement as a dependent variable and the group (A or B) as covariate. In a multivariate model, we used group, age, and sex as covariates. Finally, in a multivariate model we used the covariates group, age, sex and socioeconomic factors (SEF) including family income, education, and self-perceived financial status.

Collinearity was excluded between our independent variables and the variance inflation factor was between one and three. IBM SPSS Statistics 20.0 Software was used for data analysis. The alpha significance level was set at 0.05.

Ethics approval was obtained from the Research Ethics Board of the *Centre de santé et de services sociaux de Chicoutimi*, Quebec, Canada.

## Results

**Fig. 1:**
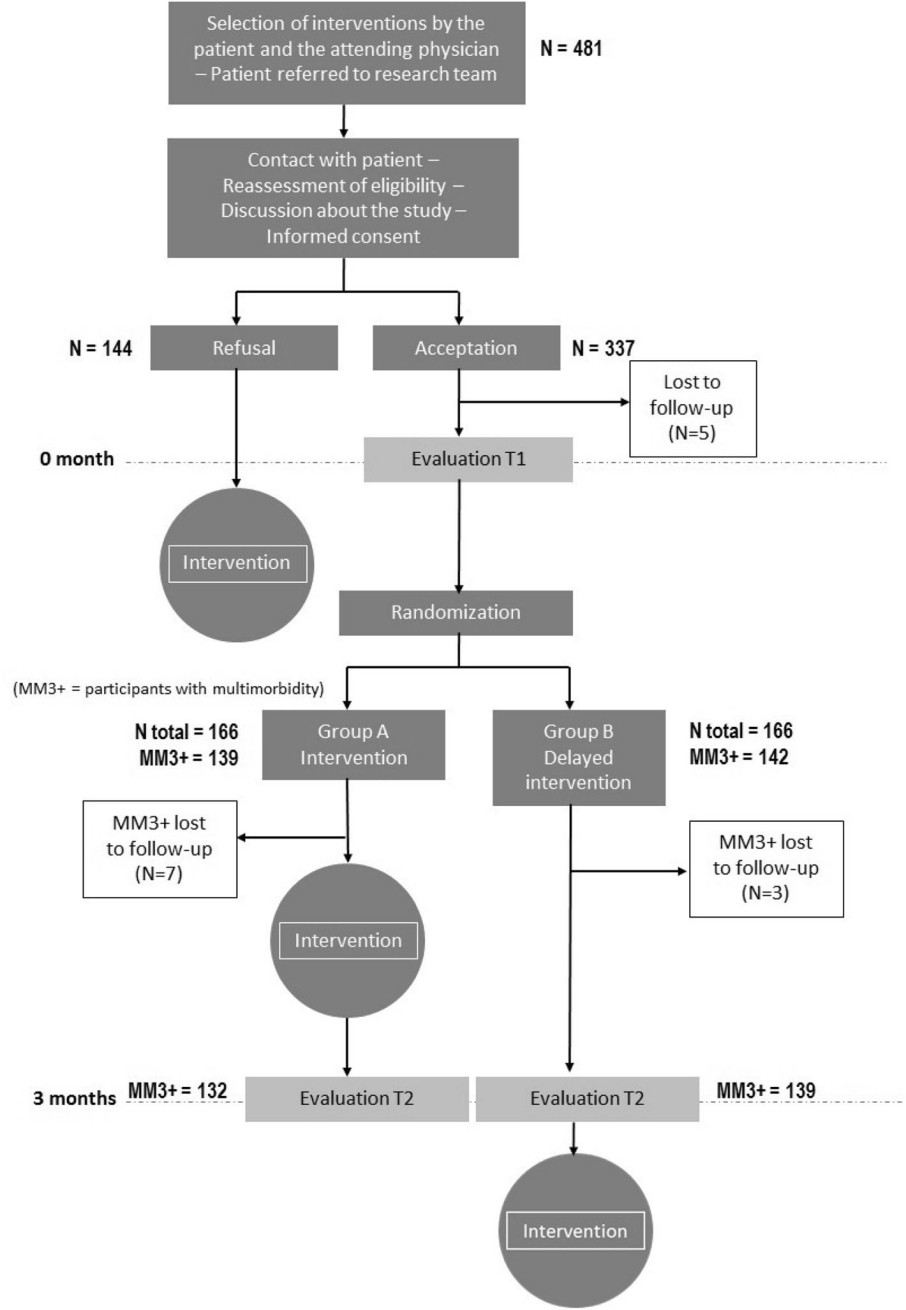
Flow diagram of the study

Fig. 1 shows the flowchart diagram of the study. The study started in November 2011 and ended in July 2012. The clinical intervention was offered to 481 eligible patients. Of these, 144 refused to participate in the research project but received the clinical intervention nonetheless. Of the 337 patients who initially agreed to participate, five were lost to follow-up before completing the baseline questionnaires. Among the remaining 332 randomized patients, there were 139 patients with multimorbidity in Group A and 142 in Group B who were the object of the present study. Seven patients from Group A and three from Group B were lost to follow-up after three months. Therefore, 132 patients from Group A and 139 from Group B underwent the evaluation after three months.

Table 1 shows the baseline demographic and clinical characteristics of participants for each group. Characteristics of the participants were similar in both groups, except for self-perceived financial status, with a higher proportion of participants in the intervention group who rated themselves as well off.

**Table 1:** Demographic characteristics of participants at baseline

	Control group *n* = 142	Intervention group *n* = 139	*p* value
Age (SD), y.	53.7 (11.3)	53.3 (11.1)	0.81
Men	72 (51%)	69 (50%)	0.86
Number of chronic diseases (SD)	5.3 (1.8)	5.7 (2.3)	0.12
Family income in CAD: < 20,000$	17 (12%)	19 (14%)	0.86
20,000-49,999$	56 (39%)	51 (37%)	
> = 50,000$	66 (46%)	65 (47%)	
Education: High school degree or less	80 (56%)	65 (47%)	0.14
Technical, college, or university	62 (44%)	72 (52%)	
Self-perceived financial status: Poor/really poor	16 (11%)	9 (6%)	0.01
Enough	79 (56%)	76 (55%)	
Well off	22 (15%)	44 (32%)	

The effects of the intervention are summarized in Table 2. Results in the intervention group were rather modest; the highest proportion of patients with a reliable improvement was observed in the domain *Skill and technique acquisition* (35.2%). The intervention was effective in six domains with odd ratios (OR) varying from 1.96 in the domain *Skill and technique acquisition* (95% CI: 1.13–3.39) to 2.91 in *Constructive attitudes and approaches* (95% CI: 1.45–5.84). In the domains, *Positive and active engagement in life* and *Social integration and support,* the ORs were not statistically significant (Table 2).

**Table 2:** Participants with a reliable improvement per heiQ domain

Domains of heiQ	Control group	Intervention group	OR (95% CI)	*p* value*
*Health-directed behaviour*	19.4% (26/134)	33.6% (43/128)	2.03 (1.16–3.55)	0.013
*Positive and active engagement in life*	13.9% (19/137)	22.1% (29/131)	1.72 (0.91–3.25)	0.092
*Emotional well-being*	14.3% (19/133)	25.6% (32/125)	1.97 (1.05–3.68)	0.035
*Self-monitoring and insight*	6.8% (9/133)	15.1% (19/126)	2.35 (1.02–5.40)	0.044
*Constructive attitudes and approaches*	9.6% (13/136)	24.2% (31/128)	2.91 (1.45–5.84)	0.003
*Skill and technique acquisition*	20.7% (28/135)	35.2% (45/128)	1.96 (1.13–3.39)	0.016
*Social integration and support*	14.0% (19/136)	16.3% (21/129)	1.14 (0.58–2.24)	0.696
*Health service navigation*	8.7% (12/138)	20.2% (26/129)	2.52 (1.21–5.21)	0.013

*Univariate logistic regression. Odds ratios (OR) and 95% confidence intervals (95% CI)

In the first multivariate logistic regression model, after adjusting for age and gender, the results remained essentially the same as the univariate model. As the domains *Positive and active engagement in lif*e and *Social integration and support* were not significantly improved in the univariate logistic regression analysis, these domains were no further tested.

In the second multivariate logistic regression model, after controlling the intervention for age, sex, and socioeconomic factors (family income, education and self-perceived financial status), the effect of the intervention on the likelihood of a reliable improvement remained significant only in four of the remaining domains of the heiQ (Table 3). The change in self-management was not significant in the domains *Emotional wellbeing* (OR: 1.93; 95% CI: 0.95–3.95) and *Self-monitoring and insight* (OR: 2.14; 95% CI: 0.84–5.42).

**Table 3:** Multivariate logistic regressions. Odds ratios (OR) and 95% confidence intervals (CI) of the effect of the intervention on the likelihood of an improvement in each domain of self-management, adjusted only for age and gender (Model 1), and adjusted for age, gender, family income, education, and self-perceived financial status (Model 2)

heiQ domain	Model 1: OR (95% CI)	*p*	Model 2: OR (95% CI)	*p*
*Health-directed behavior*	2.03 (1.16–3.55)	0.013	1.98 (1.07–3.66)	0.029
*Positive and active engagement in life* ^a^	–	–	–	–
*Emotional well-being*	1.94 (1.03–3.66)	0.040	1.93 (0.95–3.95)	0.070
*Self-monitoring and insight*	2.40 (1.03–5.57)	0.042	2.14 (0.84–5.42)	0.110
*Constructive attitudes and approaches*	2.97 (1.47–6.02)	0.002	3.92 (1.73–8.89)	0.001
*Skill and technique acquisition*	1.97 (1.13–3.41)	0.016	2.48 (1.32–4.65)	0.005
*Social integration and support* ^a^	–	–	–	–
*Health service navigation*	2.51 (1.21–5.21)	0.013	2.73 (1.20–6.22)	0.017

^a^The likelihood of an improvement in the domains *Positive and active engagement in life* and *Social integration and support* was not significantly associated with the intervention in the univariate logistic regression analysis, and therefore these domains were not tested in the multivariate analysis

## Discussion

In the search for interventions to improve outcomes in patients with multimorbidity, self-management education programs are increasingly recognized as an important component [14, 23, 24]. As multimorbidity is more prevalent in older patients and in those with lower socioeconomic status, [25] it is expected to find these characteristics among multimorbid patients included in self-management education programs.

In this present study, we observed that about one fourth of the participants who received the intervention reliably improved their heiQ scores, suggesting a modest effect of the intervention. Although the proportion of subjects in the intervention group with a reliable improvement was modest, the results are not far from those previously observed in courses for chronic diseases self-management in which, on average, one third of participants reported substantial benefits in the heiQ domains at the end of a course. [18] In the control group, we observed a number of participants who improved spontaneously in a few domains, suggesting a Hawthorne effect. [26] In addition, we should consider that patients accepting to participate in the study were volunteers and motivated to make changes to improve their health. Nonetheless, a significantly larger proportion of participants of the intervention group improved in six of the eight domains of the heiQ as compared to the control group.

Compared to the initial study (PR1MaC), our results show that patients with three or more chronic conditions improved the same domains of the heiQ (6 out of 8). The OR of improvement were comparable and slightly superior in patients with multimorbidity. Given the high prevalence of multimorbidity in primary care, it is reassuring to see that this subgroup has the potential to improve. When controlling for SEF, only four domains remained statistically significant. In one domain *(Health-directed behavior),* the OR stayed the same. In the three other domains, after controlling for socioeconomic factors, the OR improved slightly. Overall, these findings suggest that SEF has little impact on the results.

Although our study studied the effect of SEF on patients, it was not targeted specifically to a vulnerable population with low score of SEF. In an exploratory randomized-controlled study, Mercer et al. used a whole-system approach to improve the quality of life of patients with multimorbidity, targeting areas of high socioeconomic deprivation [15]. The intervention included patient-centered care and supported self-management. Quality of life improved at six months in the intervention group but was similar at 12 months. One of the three domains of well-being improved in the intervention group at 12 months. Although the results were promising, the effect of the intervention was positive in the short-term, but not significant at 12 months. This study showed promising results but also reminds us of the difficulties in developing successful interventions for multimorbid patients.

Previous studies have reported that financial constraints and low socioeconomic status in general are barriers to effective self-management. [23, 27, 28] In contrast, one study reported that controlling for education level of the participants did not influence the results of an intervention supporting self-management in patients with chronic conditions . [29] This is in contrast with a study that included patients with depression and type 2 diabetes or chronic obstructive pulmonary disease and found that education influenced the results of a self-management intervention. [30]

In this study, after adjusting for family income, education, and self-perceived financial status, in addition to age and sex, the improvement observed in the heiQ scores was no longer significant in the domains *Emotional wellbeing* and *Social integration and support*, suggesting that, at least, the results of the educational activity in these two domains are influenced by socioeconomic factors. However, there was an important overlap in the OR 95% confidence intervals before and after controlling for socioeconomic factors, suggesting a lack of power to detect the differences in this secondary analysis. More research is needed to evaluate the impact of SEF on the outcomes of interventions among multimorbidity patients. Further studies should be powerful enough to include subgroups or more vulnerable patients with low score of SEF.

### Limitations of the study

One limitation of this study is a possible selection bias. In this pragmatic intervention, the primary care providers were invited to refer patients with one of the selected chronic diseases. They may have referred only participants in whom they believed the intervention could benefit the most. All patients referred (*n* = 481) were then offered to participate in the trial. Among them, 30% (144/481) refused to participate. Altogether, these factors should have equally affected both groups.

Social desirability is a potential bias for any self-report questionnaire, but it is consistent with use for a patient-centered approach. Since participant selection could not be blinded, it is possible that part of the effect is explained by a desirability bias in the intervention group. But the heiQ has been shown to have low social desirability bias [31]. This bias was also reduced by having one designated research assistant conducting standardized interviews.

The duration of chronic diseases and multimorbidity was not assessed. Duration of disease could play a role on self-management. In one study of diabetic patients, those who had diabetes for less than two years seemed to show better improvement in self-management [32]. Also, motivation of the patients was not assessed in the initial study and could have had an impact on the results.

During the intervention, the cluster effect was limited by the fact that interventions were provided by the same health professional team and were delivered in the clinic where the subject was recruited. It is possible that external factors contributed to individual practices, but it was impossible for the research team to measure it.

A total of 481 subjects were referred to the initial study, but only 332 were randomized and 281 of them had multimorbidity. Except for age and gender, no other characteristics could be collected for those not participating. The analysis was limited to the outcome at three months. Sustainability of the changes on this subgroup of multimorbid patients is unknown.

Health literacy has been identified as potentially playing a part in explaining the link between low education and low health status [33] and it is said to play a crucial role in self-management [34]. Health literacy was not measured in this study and its effect on the results of the intervention is unknown.

It would have been interesting to receive the feed-back of the patients on how SEF affected them during the study.

## Conclusions

The self-management education intervention for patients with multimorbidity produced significant improvements in most domains of self-management. Altogether, socioeconomic factors had a minor effect on the results.

## Abbreviations

95% CI: 95% Confidence Interval
CDPM: Chronic Disease Prevention and Management
heiQ: Health Education Impact Questionnaire
OR: odds ratio
SEF: Socioeconomic factors
